# Editor's Message

**DOI:** 10.29173/jchla29636

**Published:** 2022-08-04

**Authors:** Alanna Campbell

I hope everyone has been enjoying the summer months and that this issue finds you on a sunny day – or with one on the horizon! I am excited to share with you that the *Journal of the Canadian Health Libraries Association* (JCHLA) will be available through PubMed Central (PMC) in the coming months. A big thank you to the current and previous editorial members who contributed to the PMC application process and to the board of the Canadian Health Libraries Association (CHLA) for their support.

I’d like to say a warm welcome to Meredith Fischer (Wilfrid Laurier University) who joined us in May as our new Copyeditor, and a big thank you to Amanda Caputo (Sault Area Hospital) who completed her term in April. As this is my third issue as Editor-in-Chief, it is also my final. I have truly enjoyed working with all the members of both the editorial team and CHLA board over the past three years, participating in the development and launch of JCHLA’s data sharing policy and taking steps forward with the team to address equity, diversity and inclusion through the journal’s practices and publication process. As of September 1, 2022 Colleen Pawliuk (BC Children’s Hospital Research Institute) will be Editor-in-Chief, Megan Kennedy (University of Alberta) Senior Editor and our incoming Junior Editor is Jessica McEwan (Ottawa University). Welcome Jessica!

We have a great lineup for you in this issue of JCHLA, beginning with a review of the book *Responding to Rapid Change in Libraries: A User Experience Approach*. We also have a research article from Miller and Janke and the winning 2021 student paper prize by Graham and Moleirinho. Watch for the 2022 student paper prize by Vinson Li in a future issue of JCHLA! You can also find the CHLA 2022 conference abstracts in this issue including interactive workshops, contributed papers, lightning talks and poster presentations. Thank you to all our authors and peer reviewers for your contributions!

**Alanna Campbell**

*JCHLA/JABSC Editor-in-Chief*

*Email: editor@chla-absc.ca*

J'espère que tout le monde a profité des mois d'été et que vous lisez ce numéro par une journée ensoleillée - ou une qui se profile à l’horizon ! Je suis heureuse de vous annoncer que le Journal de l'Association des bibliothèques de la santé du Canada (JABSC) sera disponible sur PubMed Central (PMC) au cours des prochains mois. Un grand merci aux membres de la rédaction, qui ont contribué au processus de demande auprès de PMC ainsi qu’ au conseil d'administration de l'Association des bibliothèques de la santé du Canada (ABSC) pour leur soutien.

J'aimerais souhaiter la bienvenue à Meredith Fischer (Université Wilfrid Laurier) qui nous a rejoint en mai, en tant que nouvelle réviseure, et remercier Amanda Caputo (Sault Area Hospital) qui a terminé son mandat en avril. Pour ma part, ce troisième numéro en tant qu’ éditrice en chef, sera aussi mon dernier. Au cours des trois années qui viennent de s’écouler, j'ai vraiment apprécié : travailler avec tous les membres de l'équipe de rédaction et le conseil d'administration de l'ABSC, participer à l'élaboration et au lancement de la politique de partage des données de l'ABSC et prendre des mesures avec l'équipe, pour aborder l'équité, la diversité et l'inclusion dans les pratiques et le processus de publication de la revue. À compter du 1er septembre 2022, Colleen Pawliuk (BC Children's Hospital Research Institute) sera éditrice en chef, Megan Kennedy (Université de l'Alberta) éditrice principale et Jessica McEwan (Université d'Ottawa), notre nouvelle éditrice débutante. Bienvenue à Jessica!

Ce numéro du JABSC vous réserve plusieurs articles de qualité, à commencer par une critique du livre *Responding to Rapid Change in Libraries : A User Experience Approach*. Nous avons également un article de recherche de Miller et Janke et le prix 2021 pour un article étudiant par Graham et Moleirinho. Surveillez l'article de Vinson Li, lauréat du prix 2022, dans un prochain numéro du JABSC ! Vous trouverez également dans ce numéro les résumés du congrès 2022 de l'ABSC, notamment les ateliers interactifs, les communications, les présentations éclairs et les affiches. Merci à tous nos auteur.es et évaluateur.rices pour leurs contributions !

**Alanna Campbell**

*Rédactrice en chef, JABSC / JCHLA*

*Courriel: editor@chla-absc.ca*

